# Guardians’ involvement in the management of childhood illnesses in Vhembe district, Limpopo

**DOI:** 10.4102/hsag.v29i0.2715

**Published:** 2024-08-20

**Authors:** Livhuwani Tshivhase, Tshifhiwa A. Magavha, Sophy M. Moloko

**Affiliations:** 1 Department of Nursing School of Health care Sciences, Sefako Makgatho Health Sciences University, Pretoria, South Africa; 2 Department of Health Studies, College of Human Sciences, University of South Africa, Pretoria, South Africa

**Keywords:** childhood illness, health seeking, guardians’ involvement, management, primary health care, qualitative study, Vhembe district

## Abstract

**Background:**

Guardians’ involvement in the management of childhood illnesses plays a pivotal role in reducing mortality and morbidity among children under 5 years old. It is through the guardian’s eyes that the child can be afforded timely healthcare, prevented from contracting an illness and effectively taken prescribed medication.

**Aim:**

The study aimed to explore the guardians’ lived experiences in their involvement in managing childhood illnesses in Vhembe district, Limpopo province, South Africa.

**Setting:**

A qualitative, exploratory and descriptive study was conducted with 16 purposively sampled participants.

**Methods:**

One-on-one individual interviews were conducted with participants. Data were analysed following Braun and Clarke’s thematic analysis.

**Results:**

Guardians reported their involvement by seeking child healthcare in healthcare facilities when ill and due for immunisations. Involvement in childhood care is performed through assessment of child illness at home, follow-up, referral of children to healthcare facilities, promotion of child health and prevention of childhood illnesses.

**Conclusion:**

Children remain dependent on guardians for their well-being. Seeking care, preventing illnesses and promoting childhood health are vital in reducing child mortality and childhood morbidity.

**Contribution:**

Involving and empowering guardians regarding the care of children under 5 years old are vital in achieving Sustainable Development Goal number 3 in 2030.

## Introduction

### Background

Childhood illnesses continue to be a significant issue in public health, especially in low- and middle-income countries with limited access to high-quality healthcare services (Murdoch et al. 2020). The Integrated Management of Childhood Illness (IMCI) strategy, initiated by the World Health Organization (WHO) and United Nations Children’s Fund (UNICEF), seeks to enhance the quality of care for children under 5 years old. The IMCI is an integrated strategy that focuses on the health and well-being of the child by reducing preventable mortality, minimising illness and disability, and promoting healthy growth and development of children under 5 years old (WHO 2024).

In addition, IMCI is a primary healthcare strategy that provides healthcare providers and guardians with the requisite expertise and understanding to handle prevalent childhood illnesses proficiently (Murdoch et al. 2020). The strategy has three components underlying its implementation: improving the skills of health workers, improving the health system and improving family and community practices. As part of the family and community practice, guardians of children under 5 years old play a pivotal role in preventing disease, early and timely identification of complications during care and maintaining healthy growth and development (WHO 2019).

In implementing the IMCI strategy, guardians are expected to be involved in managing childhood illnesses, starting by demonstrating back to the nurse how they will treat the child at home and asking for clarification regarding managing the child’s illness. Furthermore, guardians play a vital role in identifying early signs and symptoms of illness, promptly seeking medical assistance, ensuring compliance with treatment programmes and bringing children for follow-up treatment (National Department of Health 2022).

Although the IMCI strategy is being implemented in most healthcare facilities in sub-Saharan countries, there remains a notable gap in optimising the IMCI effectiveness because of suboptimal guardians’ involvement. Studies in Ghana and South Africa found that guardians’ lack of understanding and ability to identify danger signs in children under 5 years old causes delays in seeking immediate medical attention and influences the chance of children’s follow-up care (Franke et al. 2018; Pandya, Slemming & Saloojee 2018). Some caregivers prefer to buy medication at the pharmacy rather than seek child healthcare services at the clinic or hospital (Tiwari et al. 2022). The guardians’ preference for pharmacies rather than clinics was because of the guardians’ perceptions of IMCI assessment and treatment as time-consuming. Thus, children might receive incorrect medications from pharmacies because they were not assessed, which could lead to complications and unnecessary death (Reñosa et al. 2020).

On the one hand, some caregivers prefer specialists for their children’s medical care to primary or clinic care. This was attributed to their beliefs, priorities and sense of autonomy (Carai et al. 2019). On the other hand, a study in Ghana on what influences the sustainability of using child health services among guardians of children under 5 years old found that 44.2% of caregivers failed to bring children for growth monitoring, even though they knew it was necessary. Among others, the child not having completed his or her primary immunisation and poor staff attitude were the reasons for not bringing children to the facilities (Konlan et al. 2021).

Regardless of guardians’ knowledge of IMCI, a study conducted in Venda, a homeland in Limpopo province, found that parents managed diarrhoeal diseases differently. Some gave oral rehydration solutions as recommended, while some offered traditional medicine and Coca-Cola drinks (Phophi 2019). Correspondently, a similar study in South Africa found that caregivers’ practices still expose children under 5 years old to illnesses despite knowing the causes of childhood illnesses (Ndou et al. 2021). It is, however, acknowledged that some guardians often seek healthcare at the clinics when children present with symptoms such as coughing, difficulty breathing, diarrhoea, sunken eyes and convulsions (Moloko, Tshivhase & Mogotlane 2023).

It is evident from the literature that several studies have been conducted on the implementation of IMCI. However, it is unclear whether parental management of childhood illnesses may be attributed to the rurality of the Limpopo province or the access to healthcare facilities. There is still a knowledge gap regarding guardians’ involvement in managing childhood illnesses. Thus, this study aims to explore guardians’ involvement in managing children under 5 years old with illnesses at district clinics of Limpopo province.

### Purpose of the study

This study aimed to explore guardians’ involvement in managing childhood illnesses in the Vhembe district, Limpopo province, South Africa.

### Research question

How are guardians involved in the management of childhood illnesses in Vhembe district, Limpopo province?

## Research methods and design

A qualitative approach following an explorative and descriptive design was used to explore guardians’ involvement in managing childhood illnesses. A qualitative approach was followed for its ability to explore and describe or provide an in-depth understanding of human experiences such as hope or caring (Brink & Van Rensburg 2022). The design is applauded for studying human experience from the viewpoint of the research participants in the context. The study was conducted in two primary healthcare facilities: one in Musina municipality and one in Thulamela municipality in Vhembe district, Limpopo Province, South Africa. The two facilities are highly populated with children under 5 years old.

### Population and sampling

The population for the study consisted of guardians of children under 5 years old who were accessing healthcare services in the two primary healthcare facilities. All guardians who were aged 18 years and above and voluntarily consented to participate in this study were included. Participants who could communicate in local languages or English were included. The researchers purposively selected 16 guardians whom the researcher believed had experience with the phenomenon under study. The sample size was determined by saturation, which is the point wherein there were no longer new themes emerging with each interview with the study participants (Polit & Beck 2021). In this study, saturation was reached with participant number 13, but three more participants were interviewed to confirm that no new information was emerging.

### Data collection methods

Data collection methods are ways in which researchers approach answering the research question (Brink & Van Rensburg 2022). Data were collected on one-on-one interviews using a semi-structured interview guide with the participants who met the inclusion criteria. The interview guide was based on the central question of the study. Before collecting the data for the main study, three participants were interviewed to pretest the interview guide, and the collected data were not included in the main study findings. The pretest was to investigate the feasibility of the proposed study and to detect possible flaws in its methodology (Brink & Van Rensburg 2022; Gray & Grove 2021). The researchers went to the two primary healthcare facilities, secured appointments with the operational managers and agreed on the day of data collection. During the day of data collection, the researcher went to the primary healthcare facilities, approached the guardians from the clinic and recruited them as they were waiting to be seen by a professional nurse.

Information leaflets were distributed to the participants and an explanation was given of the purpose and the expectation of the study. Those who agreed to participate were asked to voluntarily sign a consent form to be interviewed and have the data published in an accredited journal. Interviews were conducted in a private room in the primary healthcare facility, which was either a postnatal room or an unused room in the facility. Participants were interviewed in English, Tshivenda and Sepedi as guided by the participant’s preferences. All the interviews were audio recorded following the participant’s consent. Each interview lasted from 30 to 45 min. Data collected in Tshivenda and Sepedi were translated into English by the researcher, who was competent in both languages commonly spoken.

### Data analysis

Data analysis was performed as described by Clarke and Braun (2006), as cited in Dawadi (2020). Audio-recorded interviews were transcribed verbatim by the researcher to facilitate the analysis process. Data transcription began within 48 h of data collection. The researcher followed the six phases of thematic analysis as: (1) familiarisation of self with the data, (2) generating initial codes, (3) searching for themes, (4) reviewing potential themes, (5) defining and naming themes and (6) producing the report.

#### Phase 1: Familiarisation

The researcher repeatedly listened to the recorded one-on-one interviews to gain an in-depth understanding of the data and acquire an overview of all collected data. This was followed by transcribing recorded interviews into written data.

#### Phase 2: Coding

Sections of the sentences were highlighted and codes were assigned to describe the content. Codes represented the notions and experiences expressed in the text. The highlighted data were grouped to identify the main points and common themes recurring throughout the data.

#### Phase 3: Generating themes

Patterns were identified among the codes and themes were derived from these patterns.

#### Phase 4: Reviewing themes

The researcher ensured that the themes accurately represented the data. A comparison was made between the identified themes and the data to verify their presence and identify any missing elements.

#### Phase 5: Defining and naming themes

Final themes were given clear, understandable names. Each theme was precisely defined, explaining what it represented.

#### Phase 6: Writing up

The data analysis was presented thematically, beginning with an introduction encompassing the research question, aims and approach. The analysis concluded by summarising the main findings and demonstrating how the analysis addressed the research question (Dawadi 2020). An independent coder was sought to analyse the data and discuss the themes with the researcher to reach a consensus over the developed themes. Table 2 presents the themes derived from the data analysis.

### Measures to ensure trustworthiness

In ensuring trustworthiness in this study, the principles were followed as described by Lincoln and Guba (1985), as cited in Brink and Van Rensburg (2022). Credibility as the truth value of data and interpretations was ensured through member checking. The researcher summarised the interpretation of the participant’s interviews immediately after each interview and then asked participants to review, validate and verify the researcher’s interpretation (Brink & Van Rensburg 2022). Confirmability, which refers to the accuracy of the data, was ensured by using an independent coder who was given the transcript to code independently and bring along themes from those transcripts. The themes developed were through the consensus between the independent coder and the researcher who collected data, which are presented in Table 2. Transferability was ensured through the thick description of the data to determine whether the study’s findings were applicable in another context or setting (Gray & Grove 2021). Dependability was ensured by keeping a detailed audit trail of the participant’s interviews.

### Ethical considerations

The study was conducted following the approval from the Sefako Makgatho University Ethics Committee (SMUREC/H/251/2023:PG) and permission from the Limpopo Department of Health, the Vhembe district manager and the operational managers of the two purposively sampled facilities. All human participants were engaged in the study in accordance with Section 13 of the Declaration of Helsinki of 1975 on observation of the ethical principles to be adhered to while conducting research with human participants (World Medical Association 2013). Informed voluntary participation was ensured with participants through verbal consent and by signing the written informed consent. Privacy and confidentiality were ensured by collecting data in a private room and using participants’ codes instead of participants’ real names when recording and writing the study report.

## Results

Sixteen participants aged 19–48 years were the sample for this study. Only 1 out of the 16 was a male guardian, while the rest were female guardians. The relationships with the children under 5 years were mothers, aunts, grandmothers and a father. Table 1 presents themes and subthemes for the study (dissertation by the second author).

**TABLE 1:** Themes and subthemes for the study.

Theme		Subtheme
1.	Guardians’ reasons for visiting healthcare facilities	1.1 Childhood immunisation service 1.2 Seeking treatment for ill health
2.	Guardians’ involvement in childcare	2.1 Managing children’s illness at home 2.2 Follow-up care in healthcare facilities 2.3 Promotion of children’s health 2.4 Prevention of childhood illnesses

Two themes with six subthemes emerged from the study’s findings. Theme one was the guardians’ reasons for visiting health facilities, which were for childhood immunisation services and to seek care for a sick child. The second theme was the involvement of the guardian in the care of the child, which was through managing children’s illness at home, follow-up care in healthcare facilities, promotion of child health and prevention of childhood illnesses. Table 2 displays the participants’ demographic data.

**TABLE 2:** Participants’ demographic data.

Participant codes	Age of participant (in years)	Gender	Number of children under guardian care	Age of the child (in months)	Participants’ relationship with the child	Facility
P01	26	F	One	36	Mother	F A
P02	48	F	Two	48 & 6	Aunt	F A
P03	19	F	One	36	Mother	F A
P04	27	F	One	20	Mother	F A
P05	20	F	One	20	Mother	F A
P06	34	F	One	24	Mother	F A
P07	42	F	One	13	Grandmother	F A
P08	28	F	One	36	Mother	F B
P09	27	F	Two	36 & 1	Mother	F B
P10	38	F	Two	48 & 1	Mother	F B
P11	33	F	Two	36	Mother	F B
P12	22	F	Two	48 & 19	Mother	F B
P13	40	F	One	36	Mother	F B
P14	37	F	One	18	Mother	F B
P15	34	M	One	24	Father	F B
P16	33	F	One	25	Mother	F A

P, participant; F, female, M, male; F A, facility A; F B, facility B.

### Theme 1: Guardians’ reasons for visiting the healthcare facilities

The study’s findings indicated that the main reasons participants visit healthcare facilities are for preventative services such as childhood immunisation or seeking treatment for ill health.

#### Subtheme 1.1: Childhood immunisation service

The study participants stated that they brought children to the healthcare facility specifically for immunisation purposes. The following quotes support the findings of the study:

‘The child has not been sick; I have brought him for immunisation only; the other one comes every month for immunisation.’ (P02, 48 years, female)

‘I usually bring the child to the clinic as per schedule in their immunisation book.’ (P05, 20 years, female)

#### Subtheme 1.2: Seeking treatment for ill health

The second common reason that participants stated as the reason for bringing their children to the clinic was ill health. These include various diseases, ailments or health problems that prompted participants to seek medical attention. The following quotes support the findings:

‘The main reason I would bring the child to the healthcare facility would be sickness. I brought the child to the clinic today because the child is coughing, vomiting, having a hot body and not eating food properly.’ (P07, 42 years, female) [*Frowns like a sad person*]

‘I don’t remember the last time I brought the child to the clinic. But I’m here today because the child is sick, swollen, and not eating.’ (P12, 22 years, female)

### Theme 2: Guardians’ involvement in childcare

Guardians are actively involved in caring for children under 5 years old by managing children’s illnesses at home, bringing them for follow-up visits to healthcare facilities, promoting children’s health and preventing childhood illness.

#### Subtheme 2.1: Managing children’s illness at home

Guardians reported participating in monitoring and managing their children’s health through assessing their children’s conditions and implementing recommended home remedies by IMCI:

‘The only thing we do when at home is only when the child has diarrhoea because that is when you know you need to follow the instruction in their immunisation book and give them the sugar, salt and water solution.’ (P04, 27 years, female)

‘I do attend to the child at home when unwell; for example, when the child is very hot and is vomiting what has been eaten, I usually bathe him in cold water to counter the fever.’ (P08, 28 years, female)

#### Subtheme 2.2: Follow-up care in healthcare facilities

Participants adhered to the advice, guidelines or treatment plans recommended during follow-up visits, demonstrating a commitment and involvement to their children’s health improvement. The excerpts supporting this are:

‘I follow the instructions I was given at the clinic on how to take care of the child. I could be giving medicines or checking whether the child is getting better.’ (P13, 40 years, female)

‘I took the child to the clinic. When I got there, the nurse gave the child medication when we were at the clinic and gave me medication to administer when I got home. She also told me to feed the child in small amounts so that the child gets some food. After administering the medication while at home, within 2–3 days, the child was well again. I also make sure that the child is being looked after at home and is always with someone.’ (P15, 34 years, male)

#### Subtheme 2.3: Promotion of children’s health

Participants indicated that they engage in health promotion activities to ensure that children are safe from contracting illness. They had these to say:

‘I make sure that the child is bathed and wears the appropriate clothes, I feed the child, I make sure that they stay indoors and avoid the cold if they are sick with flu, I also give the child food before administering medication as per instructions from the clinic.’ (P16, 33 years, female)

‘I make sure I wash hands before, and after feeding my child, and after a nappy change, I wash thoroughly with soap and water, so my child is safe from getting diseases.’ (P14, 37 years, female)

#### Subtheme 2.4: Prevention of childhood illnesses

The participants stated that they were trying their best to prevent their children from childhood illnesses. They fed the children nutritious foods to boost immunity so they would not fall sick, as evidenced by the following excerpts:

‘I make sure I breastfeed my child so that she does not get sick. I also make sure my child eats porridge and meat soup so that she can grow well.’ (P09, 27 years, female)

‘I usually feed the baby mashed vegetables because nurses tell us they are rich with vitamins which are good in preventing my child from flu. I then bathe the child, so she sleeps clean.’ (P03, 19 years, female)

## Discussions

The purpose of the study was to explore guardians’ involvement in managing childhood illnesses in Vhembe district, Limpopo province. The study’s findings revealed that guardians of children under 5 years old are involved in treating childhood illnesses. The reason for the guardians to visit healthcare facilities is to immunise their children under 5 years old. It is an expectation for a guardian to bring the child to the hospital for immunisation following the immunisation schedule in the child’s Road to Health Booklet of the child. This participation in adhering to the immunisation schedule is important to prevent the child from acquiring vaccine-preventable diseases. Immunisations and other preventive childhood measures are vital lifesaving interventions to reduce child mortality (WHO 2020). Similarly, a study in Lahore, Pakistan, found that almost two-thirds of mothers brought children to health facilities for immunisation (Kashmiri et al. 2023). Correspondingly, approximately 74% of children in sub-Saharan Africa were reported to have received the measles vaccine (Adedokun & Yaya 2020).

Although guardians in this study were determined to visit healthcare facilities for immunisation and illness of their children, another study carried out in South Africa revealed challenges among caregivers of vaccination default because of bad weather, social unrest and substance abuse (Oduwole et al. 2022). Unlike the findings of this study, some caregivers were forgetful and did not trust vaccines and missed vaccination visits (Bangura et al. 2020). Defaulting immunisations may result in disease outbreaks, consequently preventing the country from attaining the Sustainable Development Goal (SDG) number 3, which aims to reduce child mortality and morbidity by 2030 (United Nations 2021).

Furthermore, the study revealed that some guardians bring their children to the clinic when they are sick or when their condition worsens significantly, while others opt for immediate medical attention without attempting preliminary treatment at home. Most guardians were able to declare that their reason for the visit was that the child had symptoms that warranted medical treatment. Similarly, several studies conducted in Malawi, Indonesia and Nigeria attest that mothers and caregivers primarily bring their children under 5 years old to the healthcare facility when they are ill (Dougherty et al. 2020; Lungu, Darker & Biesma 2020). The most common childhood illnesses guardians seek medical care for are fever, cough and diarrhoea (Adedokun & Yaya 2020; Moloko et al. 2023).

Some participants managed childhood illnesses by assessing illnesses and providing care at home, and applying nondrug management measures such as a cold bath to reduce fever. In addition, participants gave medication to the sick child and decided when to seek care. Similarly, Prost et al. (2018) found that guardians provide home treatment for sick children, feed and give children fluids, prevent and manage child injuries and prevent abuse and neglect. Furthermore, the study conducted in Kenya affirms that guardians evaluate the child’s condition and decide to treat it at home or take the child to the clinic (Ngere et al. 2022).

The decision to treat the children at home or take them to the facilities could be based on the guardians’ knowledge about illnesses requiring healthcare services. Okunola, Aluko and Aroke (2023) asserted that caregivers knowledgeable about the various common childhood illnesses (CCI) affecting the children under 5 years old can seek care timeously. The decision of guardians to seek care is alleged to depend on a positive attitude and knowledge of the disease (Al-Noban et al. 2022). Therefore, guardians’ ability to assess the severity or see danger signs allows them to seek timely care.

Participants in this study ensured that they took the children to facilities for follow-up care as instructed by the healthcare providers. Participants further acknowledged their ability to recognise the need for immediate follow-up, especially when the condition deteriorates. In support, a study in Sudan posits that previous visits to healthcare facilities, information from family and friends, social networks and internet websites are the source of knowledge on danger signs that prompted follow-up visits (Nouman et al. 2019). These findings are consistent with the findings of this study, which indicate that previous visits to healthcare facilities are a source of follow-up.

In addition, the study participants recognised their responsibility to promote their children’s health by providing basic needs such as feeding, bathing and keeping the children safe. Consistently, a study in Limpopo province, South Africa, showed that caregivers follow growth monitoring and promotion services, as they consider them significant for the growth and development of their children (Mphasha et al. 2022). On the contrary, a study conducted in Nigeria found that caregivers lack a perfect interpretation of childhood development, health, illnesses and treatment, showing that they do not follow the instructions on the Road to Health Booklet or IMCI (Bassey, Abonor & Akintimi 2023).

Childhood development depends on guardians’ decision about the type of food the child should eat, which also depends on the availability, affordability and preferences of the child for food (Modjadji 2021). The type of food the child eats and the duration of breastfeeding affect the child’s growth (Drysdale, Bob & Moshabela 2021). Breastfeeding until 24 months, while providing nutritious age-specific supplementary feeding, improves the child’s health and growth (WHO 2021). Weaning children from breastfeeding earlier than 12 months might negatively impact child growth, consequently weakening their immunity. Low immunity exposes the child to opportunistic infections (Kashmiri et al. 2023) and could, consequently, delay the attainment of the SDG number 3 for the reduction of child mortality and morbidity by 2030 (United Nations 2021).

### Limitations

The study was conducted in two purposefully sampled primary healthcare facilities in the district, so the findings could not be generalised to the whole district. Despite this limitation, the study shared valuable information about the experiences and involvement of guardians in managing childhood illnesses.

### Recommendations

Healthcare providers should enhance communication within the healthcare system, including effective communication with guardians. Clear communication helps guardians understand their children’s conditions, treatment plans and follow-up care. Guardians must be empowered on childcare assessments and home care practices to ensure they know when and how to access childcare. Department of Health must improve health care facilities’ efficiency to reduce waiting time, which could not motivate guardians to seek healthcare.

## Conclusion

The primary reasons for guardians visiting healthcare facilities were immunisation and ill health of the child. The study also emphasised the active involvement of guardians in managing childhood illnesses, from monitoring health status to administering medication and making decisions about seeking clinic care. In summary, the research findings underscore the need for comprehensive improvements in healthcare service delivery, emphasising effective communication and guardian empowerment to enhance health for children under 5 years.
